# Seasonal variations in PM_10_ inorganic composition in the Andean city

**DOI:** 10.1038/s41598-020-72541-2

**Published:** 2020-10-12

**Authors:** Rasa Zalakeviciute, Katiuska Alexandrino, Yves Rybarczyk, Alexis Debut, Karla Vizuete, Maria Diaz

**Affiliations:** 1 Grupo de Biodiversidad Medio Ambiente Y Salud (BIOMAS), Universidad de Las Américas, Calle José Queri y Av. de Los Granados/Bloque 7, Quito, EC 170125 Ecuador; 2 Intelligent and Interactive Systems Lab (SI2 Lab) Universidad de Las Américas (UDLA), Quito, Ecuador; 3 Faculty of Data and Information Sciences, Dalarna University, 791 88 Falun, Sweden; 4 Centro de Nanociencia y Nanotecnología CENCINAT, Universidad de Las Fuerzas Armadas ESPE, Sangolquí, Ecuador; 5 Air Quality Monitoring Network, Secretariat of the Environment, Municipality of the Quito Metropolitan District, Calle Rio Coca, Quito, EC 170125 Ecuador

**Keywords:** Atmospheric science, Environmental chemistry, Environmental impact

## Abstract

Particulate matter (PM) is one of the key pollutants causing health risks worldwide. While the preoccupation for increased concentrations of these particles mainly depends on their sources and thus chemical composition, some regions are yet not well investigated. In this work the composition of chemical elements of atmospheric PM_10_ (particles with aerodynamic diameters ≤ 10 µm), collected at the urban and suburban sites in high elevation tropical city, were chemically analysed during the dry and wet seasons of 2017–2018. A large fraction (~ 68%) of PM_10_ composition in Quito, Ecuador is accounted for by water-soluble ions and 16 elements analysed using UV/VIS spectrophotometer and Inductively Coupled Plasma—Optical Emission Spectroscopy (ICP-OES). Hierarchical clustering analysis was performed to study a correlation between the chemical composition of urban pollution and meteorological parameters. The suburban area displays an increase in PM_10_ concentrations and natural elemental markers during the dry (increased wind intensity, resuspension of soil dust) season. Meanwhile, densely urbanized area shows increased total PM_10_ concentrations and anthropogenic elemental markers during the wet season, which may point to the worsened combustion and traffic conditions. This might indicate the prevalence of cardiovascular and respiratory problems in motorized areas of the cities in the developing world.

## Introduction

Particulate matter (PM) with aerodynamic diameters ≤ 10 µm – PM_10_ (including fine particulate matter ≤ 2.5 µm – PM_2.5_) are responsible for a range of health complications, varying from damages to cardiovascular and respiratory systems to premature deaths^1^. Currently, PM is one of the most challenging atmospheric pollutants due to the complexity of chemical composition related to its origin. PM_10_ can come from a variety of sources, including industries (e.g. thermoelectric power production, industrial plants, oil refinery, etc.), traffic (e.g. exhaust and non-exhaust emissions), biomass burning or wildfires, dust resuspension and chemical processes from precursor gases, among others^2–4^.

Depending on PM origin, its chemical composition, such as organic or inorganic carbon, water-soluble ions and elements, as well as its morphology can vary. A number of chemical elements such as calcium (Ca), silicon (Si), aluminium (Al) and iron (Fe) are constituents of natural soils and rocks^5^, and with magnesium (Mg), titanium (Ti) and potassium (K) have been attributed to resuspension of soil dust^6^. In addition, K has also been linked to biomass burning emissions^7^. Other metals are identified as anthropogenic tracers. For example, sodium (Na), cerium (Ce), chromium (Cr), cobalt (Co), copper (Cu), lead (Pb), lithium (Li), manganese (Mn), molybdenum (Mo), silver (Ag), zinc (Zn), zirconium (Zr), nickel (Ni) and vanadium (V), can be added during the refinement process to improve the properties of fossil fuels^8–10^. The latter two are in small amounts naturally present in petroleum-derived products^11^. A number of metals are good tracers of traffic activity—one of the biggest anthropogenic sources of PM. Fe, Zn, Cu, Pb and barium (Ba), are strong markers of exhaust emissions (Fe and Zn are used in fuel tanks’ manufacture)^12^, while Cu, Zn, Mo, Mn, Ba, Fe, antimony (Sb) and strontium (Sr), are markers of brake pads’ wear and good indicators of re-suspension dust from traffic^13^.

Apart from the sources, which may contrast conditional to the region, the concentration and chemical composition of PM can also depend on several other factors, such as meteorological parameters. While temperature and wind speed reduce PM concentrations through improved diffusion, precipitation helps scavenge air pollution through wet deposition^14–22^. It has been confirmed that the chemistry of air and precipitation simultaneously are very similar in the same location^23^. A study performed in Lanzhou, China, indicates that the scavenging effect of precipitation on different size PM depends on the strength and duration of rain events^20^. Even more, high altitude regions are exposed to distinct meteorological conditions, such as a wider-ranging daily temperature and relative humidity, higher solar and ultraviolet radiation, which boosts photochemical activity, and so forth^24^. At higher elevation, ambient temperature, air density and the oxygen available in the air are lower, which causes worsened combustion and an increase in PM emissions^25–27^.

The origins of PM have been thoroughly investigated, excluding a few regions, such as Latin America^28^. The latter is the second most urbanized region in the world^29^, with 98% of the cities violating the recommendations of the World Health Organization (WHO) for air quality^30^. Further, less is known in the high elevation cities, with a few exceptions: México City, México^31,32^; Bogotá, Colombia^34^, and Quito, Ecuador^35,36^. The highest capital in the world – Quito has a long-term air pollution problem^37–39^ resulting in reported health risks^40–42^. A previous study^37^ showed clear seasonal variations in PM_2.5_ concentrations, which have been attributed to the relative humidity and precipitation effect on combustion efficiency in this low-oxygen city. However, to better understand this seasonal contrast, the chemical composition of PM needs to be investigated. Therefore, in this work, we aim to characterize the atmospheric PM_10_, collected in urban and suburban areas in Quito, during the wet and dry seasons of 2017–2018, using Inductively Coupled Plasma—Optical Emission Spectrometry (IPC-OES). In addition, we intend to identify the difference in emitting sources during the different conditions of two contrasting seasons with the help of a correlation matrix based on a hierarchical cluster. To the best of our knowledge, this is the first work of this kind carried out in the tropical high elevation city where the characterization of PM_10_ is studied separately for seasonality. This way, the results presented herein contribute to a better understanding of the effect of meteorology on the PM composition in one of the highest elevation cities of South America.

## Materials and methods

### Site description

**Figure 1:**
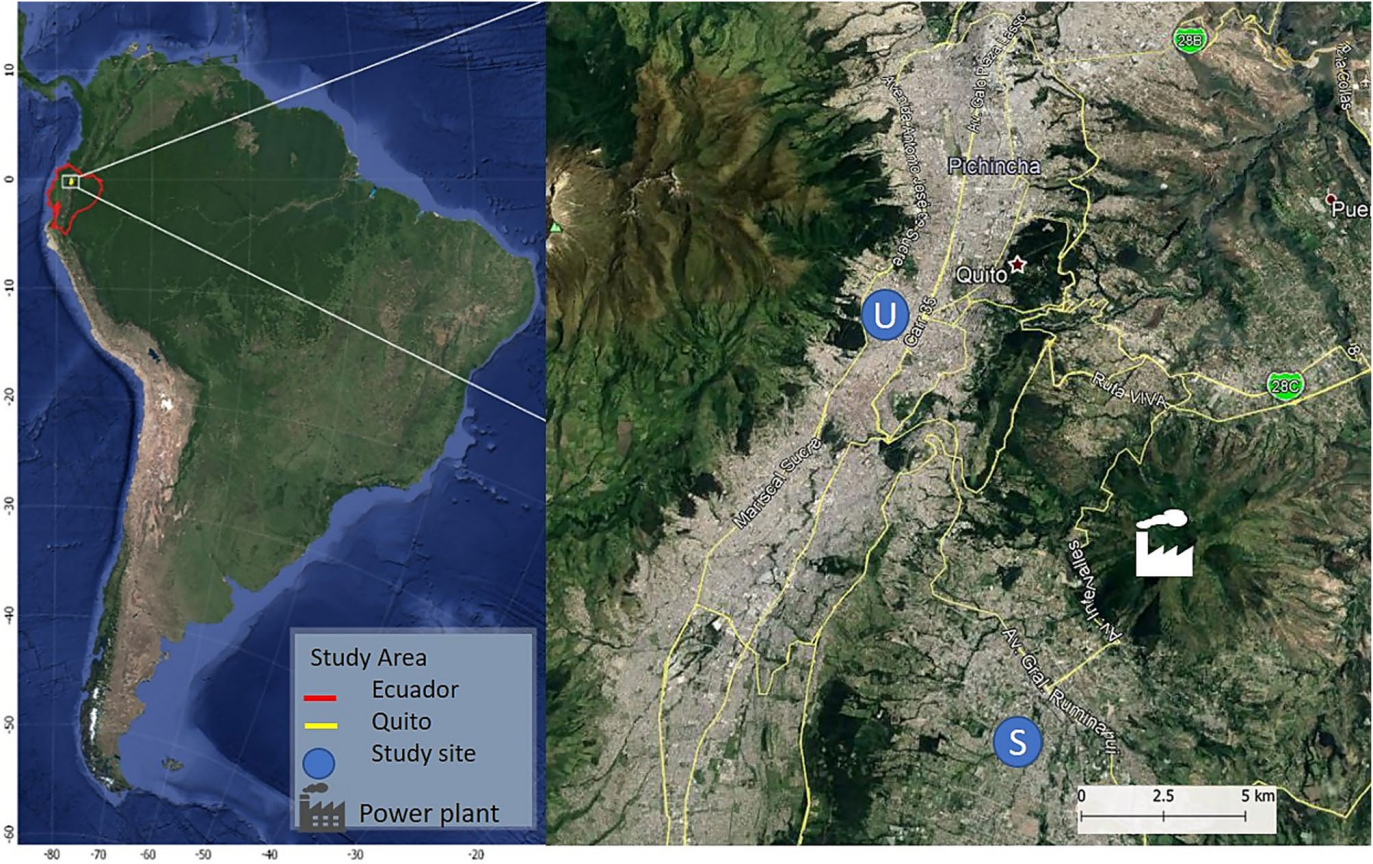
Map of the study sites in the Metropolitan District of Quito: U—central urban site and S—suburban southern valley site (blue circle markers). This figure was created using open source QGIS v.3.4 software , using QuickMapServices plugin of Google maps)^45^. https://qgis.org/.

The Ecuadorian capital—Quito, is the highest (2,850 m.a.s.l.) constitutional capital city in the world^43^. From its foundation in 1534 in the middle of the Northern Andes, the city grew to over 2,239,191 inhabitants^44^. Due to rapid expansion, it now occupies several terraces and valleys ranging from 2,300 to 3,000 m.a.s.l. on the side of an active Pichincha volcano. Quito struggles with increasing rush-hour problems, worsened by the lack of space in a very complex topography (Fig. 1). On top of that, the region uses poor quality fuels, which, with an increasing motorized fleet, causes a deteriorating air quality^39^. High direct sun radiation, combined with high elevation, contributes to persistent photochemical smog events. Tropical Andean Quito is characterized by a clear definition of rainy (September–May) and dry (June–August) seasons^37^.

The PM_10_ sampling was performed at two sites in the city (Fig. 1). A central urban site (elev. 2,835 m.a.s.l., coord. 78° 29′ 24″ W, 0° 10′ 48″ S) is located in the most urbanized part of the city. This central area of the study contains a busy network of streets, avenues and a highway. A southern valley suburban site (elev. 2,453 m.a.s.l., coord. 78° 27′ 36″ W, 0° 18′ 00″ S) is located in a less urbanized residential area.

### Atmospheric pollution and meteorological data

High-volume PM_10_ samplers (Tisch Environmental, INC.; EPA reference method) were employed to trap particulate matter with aerodynamic diameters equal or under 10 µm on quartz fibre filters (Whatman, 20.3 cm × 25.4 cm). High-volume PM_10_ samplers were coupled with the complete criteria pollution (NO_x_, SO_2_, CO, O_3_, PM_2.5_) and meteorological (temperature, solar radiation, wind speed, precipitation and relative humidity) stations, described previously in another study^37^. The monitoring stations were erected on the elevated terraces of buildings, at about 10 m from the ground level at both—the urban and suburban—experimental sites. The High-volume PM_10_ samplers were operating at an approximate 1.1 m^3^ min^−1^ flow rate under local temperature and pressure conditions. This flow rate was later corrected at EPA standard conditions of temperature (25 °C) and pressure (760 mmHg)^46^.

Samplings of PM_10_ and complementary parameters were carried out between January 1st of 2017 and December 30th of 2018 during the dry and rainy seasons. The 24-h filter samples of particulate matter were collected every six days during the study period. This sampling frequency has been largely used by other authors^47–49^, and it has been shown that it is a good frequency to determine the seasonal variation in the PM concentration and chemical composition^50,51^. To minimize costs, two filters per month for each site were selected to carry out the chemical analysis. Thus a comparative analysis was performed between the average concentration data of PM_10_ filters and the “maximum potential” 24-h data from a reference site located 6–15 km north (using automatic Thermo Scientific/FH62C14 (PM10 EPA No. EQPM-1102-150) equipment) (see Fig. A1, Online Appendix A). It can be observed that PM_10_ filter data have a good representativity of the time series of significantly higher resolution PM_10_ concentrations.

Complementary atmospheric chemistry and meteorological data, collected at an hourly time-step, were organized by 24-h averages, corresponding to the PM_10_ sampling days, with an exception of daily (and annual) accumulation of precipitation, and then averaged by seasonality (Microsoft Office Excel software). As common in the tropics, there is a differentiation between the dry and rainy seasons. In addition to looking at the atmospheric chemistry differences during the seasons, we split the rainy season data into days with and without rain events. This is important as rain might have a cleansing effect on atmospheric pollution, but elevated relative humidity during rainy season, even without rain events, may also play an important role in PM chemistry. Figures were made using MS Excel v.10^52^ (Office 365), Igor v.8.0 (WaveMetrics)^53^ or open source Rstudio^54^ v.3.1 softwares (https://rstudio.org). The map of the study area was created using an open source QGIS 3.4 software https://qgis.org/, using QuickMapServices plugin for Google maps)^45^.

### Filter conditioning and weighting procedure

Before and after the sampling, the quartz fibre filters were conditioned for 48 h in a room at a constant temperature (20 ± 3 °C) and relative humidity (50 ± 5%). The conditioned filters were weighted in a ± 0.01 mg sensitivity microbalance (Radwag). Five consecutive weight determinations were performed. After sampling, the filters were stored in zip-lock bags kept at 4 °C to prevent the volatilization of matter from the filter until chemical analyses were performed. The net particulate mass gain was determined gravimetrically by the weight of the filter before and after sampling.

### Chemical and morphological characterization of PM_10_

PM_10_ filters were analysed for the chemical and morphological composition of PM_10_. A total number of 90 samples (45 per site) were used for elemental analysis using Inductively Coupled Plasma—Optical Emission Spectroscopy (ICP-OES, Thermo Scientific iCAP 7000 Series). This equipment is calibrated with ICP-OES standards and is able to detect 30 elements (B, Ba, Bi, Cd, Co, Cr, Cu, K, Mn, Ni, Pb, Sr, Ti, Zn, Ca, Fe, Al, Na, Li, Ag, Ga, Mg, V, Te, Se, S, P, Si, As, and Be).

Dissolution of loaded and blank filters (approximately 0.0016 m^2^) was performed by microwave heating in closed Teflon vessels with 10 mL of nitric acid (65% v/v) at 200 °C for 45 min. The extract was finally filtered and diluted with ultrapure water to a final volume of 50 mL.

The recovery percentage of the extraction method was evaluated using the NIST1648a – Urban particulate matter reference material (Sigma-Aldrich). Table 1 shows the recovery percentage (> 85%) for 16 elements (explaining ~ 92.6% of elemental mass for the total mass of 30 analysed elements), as well as, the limit of detection (LOD) and quantification (LOQ) for each element, which were calculated as in Ref.^35^. The data of these elements were therefore used in this study: Cd, Cr, Cu, K, Mn, Ni, Pb, Sr, Zn, Ca, Fe, Al, Na, Mg, V and S.

**Table 1:** Limits of detection (LOD) and quantification (LOQ) of ICP-OES measurements and the recovery percentage.

Element	LOD (µg g^−1^)	LOQ (µg g^−1^)	Certified value (µg g^−1^)	Found value (µg g^−1^)	Recovery (%)
Cd	0.00024	0,00,082	73.70	76.52	103.83
Cr	0.00021	0.00071	402	407.14	101.28
Cu	0.0055	0.018	610	582.80	95.54
K	0.010	0.035	10,560	9,976,90	94.48
Mn	0.0014	0.0046	790	757,198	95.85
Ni	0.0018	0.0061	81.10	74.34	91.66
Pb	0.0032	0.011	6,550	6,574.90	100.38
Sr	9.66E-06	3.22E-05	215	184.21	85.68
Zn	0.016	0.054	4,800	4,399.07	91.65
Ca	0.00018	0.00060	58,400	49,996.84	85.61
Fe	0.0062	0.020	39,200	34,364.14	87.66
Al	0.022	0.073	34,300	32,412,54	94.50
Na	0.026	0.086	4,240	4,515,86	106.51
Mg	0.0013	0.0045	8,130	7,370,11	90.65
V	0.00031	0.0010	127	109.4	86.15
S	0.84	2.79	55,100	53,006.74	96.20

The same set of 90 filters run for elemental analysis were used to determine the fraction of water-soluble ions (NO_3_^−^, NH_4_^+^ and SO_4_^2−^) in PM_10_. Another 0.0016 m^2^ of the filter was processed in an Erlenmeyer flask with 100 mL of Type I water using an ultrasound bath for 1 h and then filtered through the Whatman filter paper. For nitrate analysis, 25 mL of sample was placed with a 1 mL of 1 N Hydrochloric acid for 20 min. After the reaction, it was read at 220 nm in the UV/VIS spectrophotometer (Shimadzu brand)^55^. For ammonium analysis, a 25 mL of sample was processed with 1 mL of Phenol Solution, 1 mL of 0.5% Sodium Nitroprusside Solution and 2 mL of Alkaline Citrate and Sodium Hypochlorite. After 1 h, it was evaluated by the UV/VIS spectrophotometer at 640 nm (Shimadzu brand)^56^. For sulfate analysis, 25 mL of sample was added to 5 mL of Buffer A solution (30 mg MgCl_2_·6H_2_O, 5 g CH_3_COONa·3H_2_O, 1 g KNO_3_, 20 mL CH_3_COOH diluted in a total volume of 1,000 mL) and 0.5 g of pure Barium Chloride. Then the sample was stirred for 1 min and analysed using the UV/VIS spectrophotometer at 420 nm (Shimadzu brand).

From the 90 PM_10_ filter samples selected for the chemical analysis, 20 samples were selected to carry out the morphological analysis using Scanning Electron microscopy (SEM). Out of 10 filters per each site, 5 filters were selected to represent the dry season and 5 filters the wet season. The morphological analysis was elaborated using a Tescan Mira 3 microscope equipped with a Schottky Field Emission Gun (FEG-SEM) that allows us to get a resolution of 1.2 nm at 30 keV. The sample analyses were performed by fixing small fragments of cinders of around 0.125 cm^3^ on SEM stubs and covering them with 20 nm of a conductive gold layer (99.99% purity) using a sputtering evaporator Quorum Q150R ES.

SEM images of PM_10_ filter samples for urban and suburban sites in Quito were further categorized based on different meteorological conditions: (1) No/low rain during the wet season (0–1 mm/24-h); (2) Medium rain during the wet season (> 1–9 mm/24-h); (3) Strong rain during the wet season (> 9 mm/24-h); and (4) No rain during the dry season. These thresholds were based on previous studies^21,37^.

### Clustering analysis

A hierarchical clustering (HC) algorithm was used to build a correlation matrix heatmap. Our script was based on an agglomerative clustering algorithm, called AGNES (AGglomerative NESting), which implements a bottom-up method. That is, each observation is initially considered as an individual cluster. Then, at each iteration of the program, the two most similar clusters are integrated into a new bigger one. This process pursues until all the observations are grouped into a single large cluster (root of the tree). The library “cluster” in R is used to develop such a script.

As in any clustering technique, the (dis)similarity between the instances is measured through a distance metric, which is commonly a Euclidian distance. In addition, a cluster agglomeration method (also known as the linkage method) is used to measure the distance between two clusters. Here, the linkage method was based on the Ward technique, which consists in minimizing the total within-cluster variance. At each iteration, the pair of clusters with the smallest between-cluster distance are merged. This technique is preferred over the other ones (average, single, complete, or centroid linkage clustering) because it is the most suitable for AGNES and it tends to produce more compact clusters^57^.

A heatmap correlation matrix is generated in open source RStudio v1.3 (https://rstudio.com) from the agglomerative HC and using the library “corrplot”. We first compute the dissimilarity values (Euclidian distance) and, then, feed these values into the function corrRect.hclust() by specifying Ward as the agglomeration method. The script defines the best clustering of metals (elements that are highly and positively correlated are merged into the same cluster) according to the number of clusters requested. These clusters were visualized on the heatmap through squares highlighted in bold.

Here, six correlation matrices HC (CMHC) were run: two studied sites (urban and suburban) for every one of the three climatic conditions (dry season, wet season with rain days and wet season without rain days). 26 variables were analysed: 16 elements (Na, Ca, Al, Fe, Mg, Mn, Ba, Ni, V, Cr, Cu, Zn, Pb, Cd, Sr and S), three water-soluble ions (NO_3_^−^, NH_4_^+^ and SO_4_^2−^), two diameters of particulate matter (PM_2.5_ and PM_10_), and five meteorological factors (wind speed, relative humidity, precipitation, solar radiation and temperature). The analysis was based on 16 records for the dry and wet season without precipitation. 12 records were available for the analysis of the wet season with rain. The resulting CMHC allows us to emit hypotheses regarding the pollution source of the metals based on the difference of the composition of each cluster (natural vs. anthropogenic origin) according to the locations (urban vs. suburban) and the climatic conditions (dry vs. wet season).

## Results and discussion

### Seasonality in concentrations of PM_10_ and its chemical composition

**Figure 2:**
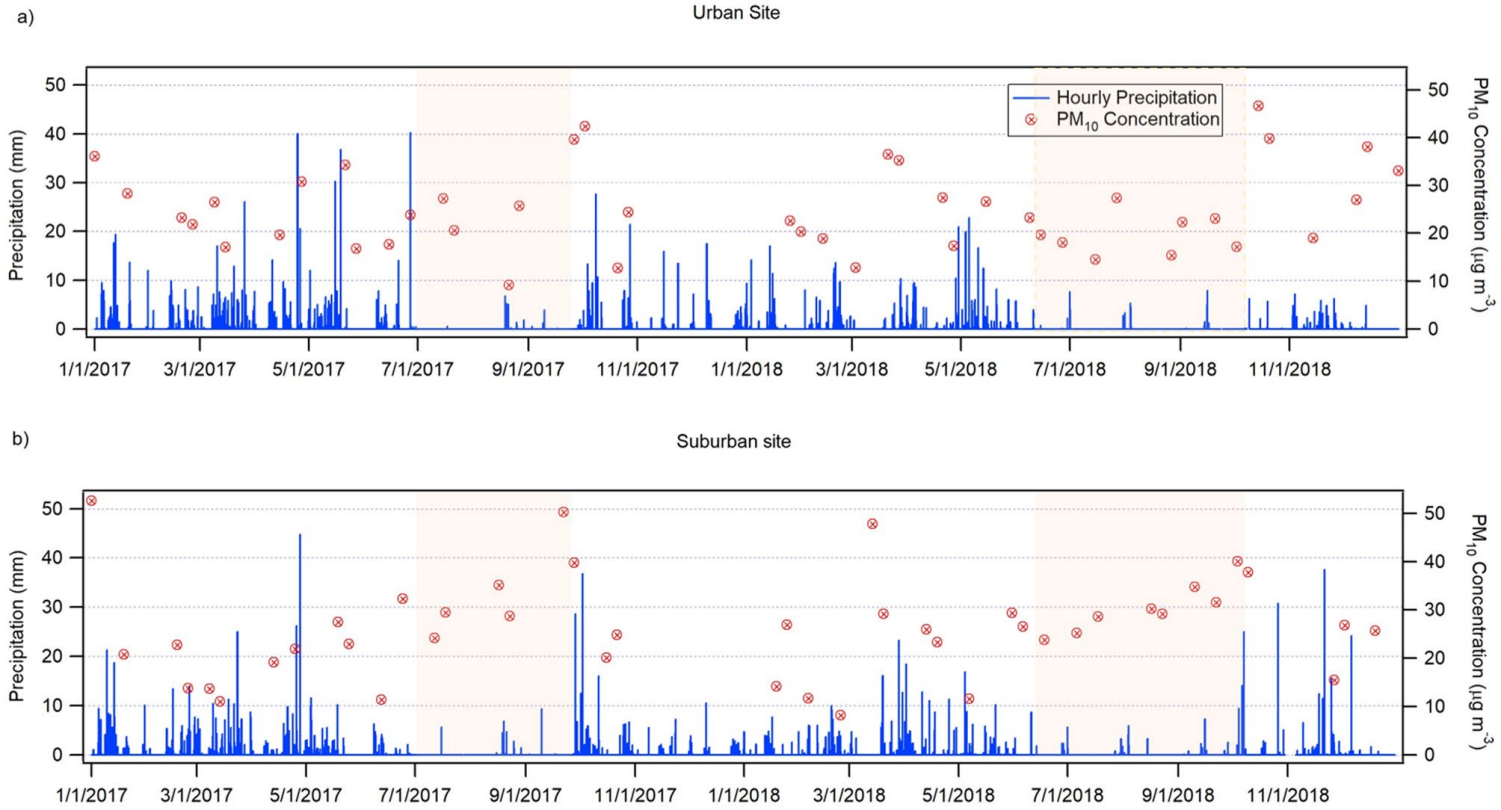
Hourly cumulative precipitation and 24-h PM_10_ concentrations at urban and suburban sites during 2017 and 2018 (the red shaded areas indicate dry seasons). This figure was produced using Igor Pro (WaveMetrics, Inc) v.8.0 software^53^.

24-h PM_10_ filter concentrations at the urban and suburban sites during 2017 and 2018 are presented in Fig. 2. This data is accompanied by the hourly cumulative precipitation data. It can be observed that the PM_10_ concentrations at the urban site tend to increase during the wet season and at the suburban site during the dry season (identified by the red shaded areas, Fig. 2). To help better understand the distinctions of these results, Table 2 summarizes the statistics of the concentrations of PM_10_ and its chemical elements. In addition, Table 3 shows the statistics of water-soluble ion concentrations, and Table 4 provides the statistics of meteorological parameters and criteria pollutant concentrations. All these statistics are organized by the distinct periods: (1) dry season; (2) wet season without rain days; and (3) wet season with rain days. Table 2 specifies that at the urban site the highest PM_10_ average concentrations are registered during the wet season days without rain events, as also most of the elements. This result agrees with other studies^58^. This increase in the concentrations of PM_10_ and elements during the wet season may be due to an increase in relative humidity (see Table 4), which causes worsened combustion efficiency^37^; urban dynamics, such as an increase in the acceleration and braking rates, which can raise the pollutant emission rate^25,58^; and a higher intensity of anthropogenic activity during school period (September–June). The latter causes an increase in the use of highly contaminating diesel-powered school buses^39^. The increased PM_10_ concentrations during the wet season without rain events can be associated with the combination of a high RH, which can promote the formation of particles in the absence of rain events, thus lack of wet scavenging^59^. Meanwhile, at the suburban site, the increased PM_10_ levels are registered during the dry season, confirming the findings of other studies^60,61^. This can be attributed to the increase in solar radiation during the dry season, that augments temperature and wind speed, and, in turn, causes more particle resuspension^62–65^, additional to a lack of wet scavenging.

**Table 2:** Seasonal variation (dry season, wet season without rain events and wet season with rain events) of average concentrations ± standard deviation of PM_10_ in filters and chemical elements (Ca, Na, S, Mg, K, Fe, Al, Zn, Cu, Cr, Sr, Mn, Pb, Ni, V, Cd) for the urban and suburban sites during 2017 and 2018.

Element	Urban site			Suburban site		
	Dry	Wet without rain	Wet with rain	Dry	Wet without rain	Wet with rain
PM_10_ (µg/m^3^)	21.15 ± 9.96	**29.22 ± 9.52**	24.53 ± 7.69	**29.03 ± 8.00**	24.46 ± 10.46	24.61 ± 12.75
**µg/m**^**3**^						
Ca	8.38 ± 3.08	**8.46 ± 2.10**	7.65 ± 2.13	7.34 ± 3.00	**7.52 ± 1.43**	7.31 ± 1.64
Na	4.56 ± 2.09	**5.53 ± 2.29**	4.76 ± 1.39	4.45 ± 2.64	**4.95 ± 1.53**	4.32 ± 1.04
S	1.46 ± 0.72	**2.47 ± 0.98**	2.21 ± 0.54	1.63 ± 0.86	**2.36 ± 0.86**	2.10 ± 0.98
Mg	1.98 ± 1.02	**2.06 ± 0.66**	1.89 ± 0.70	1.70 ± 0.96	**1.90 ± 0.59**	1.81 ± 0.52
K	0.73 ± 0.71	**0.84 ± 0.58**	0.63 ± 0.30	0.55 ± 0.30	**0.71 ± 0.42**	0.65 ± 0.25
Fe	0.42 ± 0.16	**0.61 ± 0.17**	0.46 ± 0.18	**0.48 ± 0.20**	0.47 ± 0.28	0.39 ± 0.23
Al	1.24 ± 2.04	**2.06 ± 1.71**	1.42 ± 1.77	1.52 ± 1.97	**1.71 ± 1.91**	1.47 ± 1.57
**ng/m**^**3**^						
Zn	93.32 ± 107.88	85.31 ± 60.18	**105.18 ± 74.68**	93.77 ± 125.64	104.72 ± 67.84	**113.24 ± 86.34**
Cu	75.84 ± 67.29	109.32 ± 83.88	**112.47 ± 87.91**	**160.80 ± 230.24**	142.13 ± 122.71	105.42 ± 55.40
Cr	15.54 ± 21.32	**88.12 ± 287.22**	38.16 ± 84.91	5.92 ± 3.70	**11.39 ± 8.36**	9.28 ± 7.06
Sr	55.90 ± 38.57	**60.96 ± 37.33**	52.82 ± 28.94	38.93 ± 31.92	48.17 ± 22.71	**51.86 ± 28.01**
Mn	49.33 ± 20.10	**51.83 ± 14.20**	45.41 ± 17.56	42.44 ± 25.00	**47.80 ± 16.68**	47.63 ± 16.25
Pb	16.06 ± 28.28	**29.08 ± 36.63**	14.10 ± 13.39	13.52 ± 11.59	**38.28 ± 38.78**	15.29 ± 19.82
Ni	11.04 ± 31.31	**18.25 ± 23.15**	9.05 ± 7.21	5.74 ± 6.96	**16.61 ± 10.10**	13.89 ± 13.32
V	6.13 ± 4.44	**28.46 ± 47.68**	10.23 ± 4.84	11.86 ± 5.71	**34.32 ± 26.23**	28.59 ± 43.67
Cd	34.14 ± 55.66	**43.08 ± 76.13**	26.80 ± 44.42	22.95 ± 43.74	12.31 ± 27.23	**25.20 ± 40.41**

The highest average values are shown in bold.

**Table 3:** Seasonal variation (dry season, wet season without rain events and wet season with rain events) of average concentrations ± standard deviation of water-soluble ions (NH_4_^+^, NO_3_^−^ and SO_4_^2−^) fraction of PM_10_ in filters, for the urban and suburban sites during 2017 and 2018.

Ion (µg/m^3^)	Urban site			Suburban site		
	Dry	Wet without rain	Wet with rain	Dry	Wet without rain	Wet with rain
NH_4_^+^	0.07 ± 0.06	0.07 ± 0.05	**0.16 ± 0.21**	0.05 ± 0.04	**0.07 ± 0.07**	0.04 ± 0.026
NO_3_^−^	0.27 ± 0.05	**0.45 ± 0.19**	0.38 ± 0.15	0.31 ± 0.16	**0.39 ± 0.13**	0.38 ± 0.10
SO_4_^2−^	2.66 ± 1.53	2.83 ± 1.25	**3.49 ± 1.31**	1.89 ± 1.16	**2.49 ± 1.13**	2.25 ± 1.51

The highest average values are shown in bold.

**Table 4:** Seasonal variation (dry season, wet season without rain days and wet season with rain days) of average concentrations ± standard deviation of atmospheric pollutants (CO, NO_2_, O_3_, SO_2_ and PM_2.5_) and meteorological parameters [relative humidity (RH), precipitation (Prec), temperature (T), solar radiation (SR), wind speed (WS)] for the urban and suburban sites during 2017 and 2018.

Parameter	Urban site			Suburban site		
	Dry	Wet without rain	Wet with rain	Dry	Wet without rain	Wet with rain
RH (%)	54.09 ± 10.04	65.83 ± 18.18	**82.12 ± 23.61**	58.44 ± 9.66	71.68 ± 18.66	**76.98 ± 22.11**
Prec (mm)	0.79 ± 3.18	0 ± 0	**9.73 ± 8.88**	0.12 ± 0.48	0.01 ± 0.03	**6.72 ± 7.05**
T (°C)	**14.90 ± 0.65**	14.49 ± 3.74	13.06 ± 3.72	**16.53 ± 0.64**	16.14 ± 4.01	15.39 ± 4.34
SR (W/m^2^)	**948.44 ± 88.42**	884.24 ± 140.28	630.94 ± 118.95	**934.38 ± 81.61**	847.38 ± 136.52	820.44 ± 148.97
WS (m/s)	**1.89 ± 0.59**	1.40 ± 0.53	1.09 ± 0.34	**1.67 ± 0.51**	1.17 ± 0.34	1.2 ± 0.46
CO (mg/m^3^)	0.51 ± 0.16	0.61 ± 0.24	**0.72 ± 0.27**	0.58 ± 0.14	0.53 ± 0.16	**0.62 ± 0.23**
NO_2_ (µg/m^3^)	22.79 ± 5.31	**27.25 ± 13.03**	26.73 ± 26.73	19.54 ± 3.99	18.53 ± 6.69	**21.31 ± 11.28**
O_3_ (µg/m^3^)	**27.61 ± 10.39**	19.49 ± 8.94	15.38 ± 5.66	**28.28 ± 11.15**	24.08 ± 8.94	23.72 ± 9.64
SO_2_ (µg/m^3^)	2.52 ± 1.07	**4.18 ± 3.78**	3.13 ± 1.42	4.37 ± 1.27	5.62 ± 5.79	**7.06 ± 7.18**
PM_2.5_ (µg/m^3^)	11.59 ± 3.79	17.04 ± 7.11	**17.90 ± 6.67**	13.19 ± 3.86	16.04 ± 10.18	**17.16 ± 11.78**

The highest average values are shown in bold.

Regarding the chemical element concentrations, at the urban site, all the elements display higher concentrations during the wet seasons, while at the suburban site, the concentrations of Fe and Cu are higher in the dry season (Table 2). This indicates that the increase in PM_10_ concentrations at the urban site during the wet season could be associated with an accumulation of pollution from natural and anthropogenic activities (such as traffic-related accelerating and braking activities). This is expected because the urban site is in an area with high vehicular flow. Meanwhile, at the suburban area the increase in PM_10_ concentration during the dry season could be associated with the natural sources, such as re-suspension of dust from roads (e.g., higher wind speed, Table 4). While, Cu and Fe are naturally present in soils, they also might be transported from the neighbouring industrialized area^35^ with metallurgic industries or traffic^28,66^ during the windier dry season.

Similar to the total PM_10_ and elemental composition, water-soluble ion concentrations also display an increase during the wet season at both sites (see Table 3). This is due to the increase in humidity, the decrease in temperature and weaker winds during the wet season (see Table 4) that help the transformation of ions from gas to particle phase^67^. The highest ion concentrations are found at the urban site. Moreover, increased levels are registered during the wet season without rain events at both sites. An exception is for SO_4_^2−^ and NH_4_^+^ concentrations at urban site, where the highest concentrations of these ions are found during the wet season with rain events. The higher SO_4_^2−^ concentration during the wet season with rain events at the urban site could indicate that, during this period, this ion was originated from the local emission sources such as coal and diesel combustions and could also be related to the lower rate of removal via wet deposition^68^. Moreover, as it is reported in the literature, most of the existing NH_4_^+^ is bounded to SO_4_^2−^ to form ammonium sulfate (NH_4_)_2_SO_4_^69^, thus the higher NH_4_^+^ during this period is related to the higher SO_4_^2−^ concentration. At both sites, the lower ion concentrations during the dry season, compared to the wet season, may be due to the volatilization of ammonium nitrate (NH_4_NO_3_) and ammonium sulfate, the latter being more stable at higher temperatures (less volatile) than the former^70,71^. This explains the higher concentration of SO_4_^2−^ in particles during the wet season.

### Seasonality of complementary data

The urban site displays a higher average accumulation of daily precipitation and a bigger variation in RH between the seasons (54.09–82.12%) compared to the suburban site (58.44–76.98%) (Table 4). Table 4 shows that RH increases at both sites during the wet season, especially during the days of rain events. There is a small variation in daily average temperatures between the seasons, as the solar angle varies very little during the year (66.5°–90°), and most of the precipitation is convective (solar heating), forming in the afternoons. As a result, the wet season with rain events has the lowest daily average temperatures with a high standard deviation. The dry season can be characterized by increased solar radiation, resulting in increased ozone formation and augmented wind speeds in the city (Table 4). The latter also points to an increased resuspension of particulate matter^65^, especially evident at the suburban site (less paved surfaces). All the other criteria pollutants display an increase in average daily concentrations during the wet season. Specifically, CO and PM_2.5_ show the highest levels during the wet season days with rain events, when the traffic conditions worsen, as it was previously described in another study^37^. Moreover, the increase in the concentrations of atmospheric pollutants (except for ozone) during this period is also due to the adverse meteorological conditions (high RH, low temperature and low WS) that increase the emission and the accumulation of these pollutants^72^. These findings help explain the peak concentrations of most of the chemical components at both sites, except for the natural markers in the suburban site (Table 2).

### Clustering analysis

**Figure 3:**
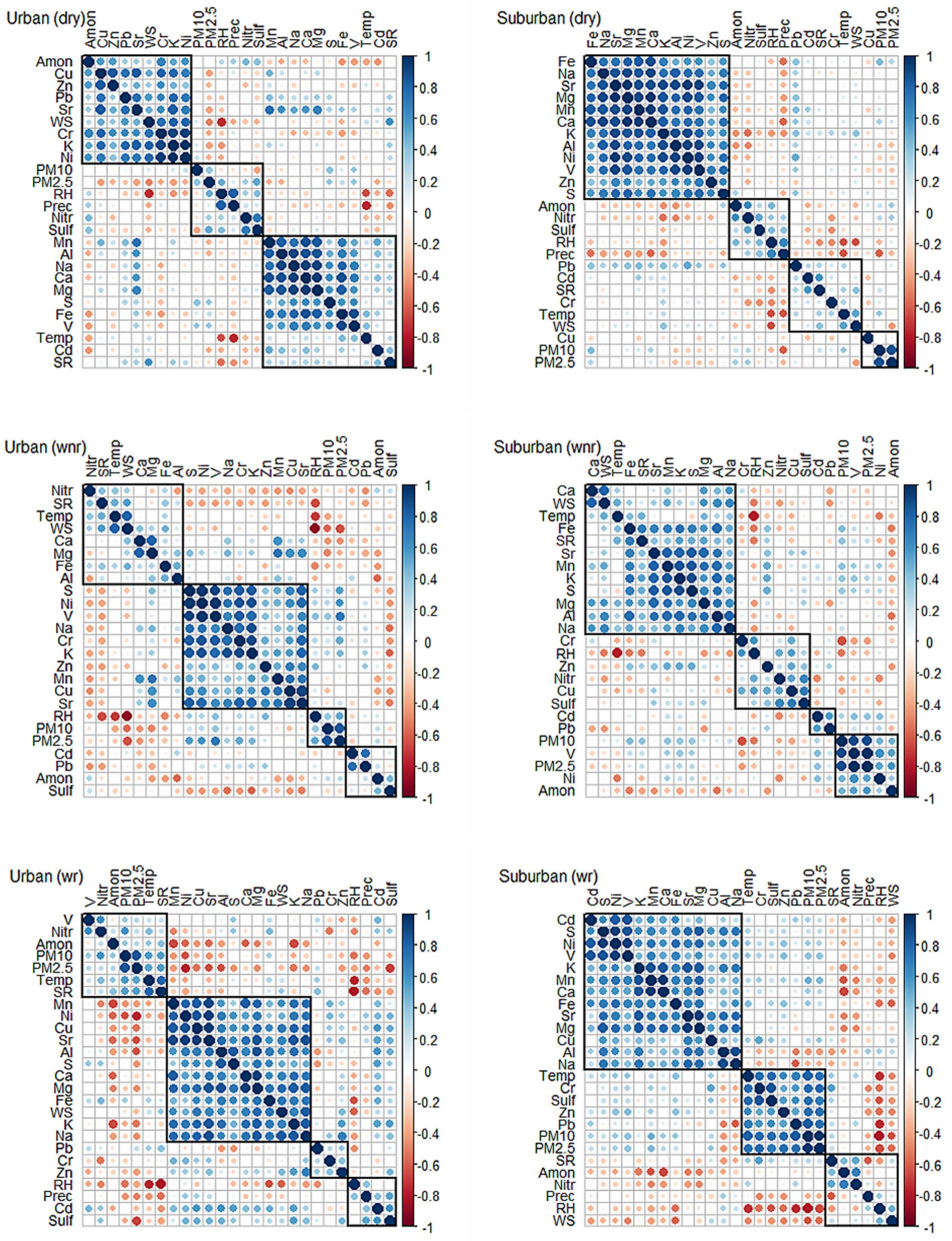
Seasonal [dry season (dry), Wet season days without rain (wnr) and Wet season days with rain (wr)] linear correlation and cluster analyses between meteorological parameters [relative humidity (RH), precipitation (Prec), wind speed (WS), temperature (Temp), solar radiation (SR)] and the concentrations of PM_2.5_, PM_10_ and its chemical elements and ions (NH_4_^+^ (Amon), SO_4_^2−^ (Sulf) and NO_3_^−^ (Nitr)) for urban (**a**–**c**) and suburban (**d**–**f**) sites during 2017–2018. This figure was prepared using open source Rstudio v.3.1 software ()^54^. https://rstudio.com.

Figure 3 presents the linear correlation and cluster analyses between the meteorological parameters (RH, precipitation, WS, temperature, SR) and the concentrations of PM_2.5_ and PM_10_ and its chemical composition (elements and water-soluble ions). It shows that, at the central urban site, during the dry (windier) season (Fig. 3a), Cu, Zn, Pb, Cr, K, Ni and Sr strongly positively correlate with WS. This suggests that these, mostly industrial metals, are transported to this site from the more industrial parts of the city. Furthermore, Mn, Al, Na, Ca, Mg, S, Fe, V and Cd also correlate with SR, which in turn strongly correlates with WS, also suggesting the resuspension and transport of these elements. This is indirectly confirmed at the suburban site (Fig. 3d), where most of those above-mentioned elements highly correlate with each other. Meanwhile, an increase in RH and precipitation helps reduce PM_10_, but it increases PM_2.5_, with an exception for the suburban site where precipitation reduces particulate pollution. The latter has been reported by a previous study^37^, highlighting different precipitation effects in urban and suburban areas. These findings are supported by Table 4, where higher concentrations of all criteria pollutants are registered during the wet season at the urban site, with an exception of O_3_—a result of photochemical activity. On the other hand, during the wet season, the concentrations of natural markers (e.g., Mn, Na, Ca) increase with an increase in SR, T and WS, or with a decrease in RH. When RH increases, the levels of natural elements generally decrease (Figs. 3b,c,e,f). Meanwhile, when RH increases, the concentrations of anthropogenic-origin elements (e.g., S, Ni, V, Cr, Zn, Cd, Pb) increase during the wet season without rain events due to a worsened combustion efficiency. However, they tend decrease during the wet season with rain events, due to wet deposition and the dry season due to increased ventilation, at both sites.

Regarding the water-soluble ions, ammonium has a different trend from sulfate and nitrate during the dry season at the urban site. Whereas, the ammonium correlates with anthropogenic elements and wind speed (Fig. 3a), suggesting its transport to the urban area from the city outskirts, in the suburban area it shows an opposite effect, where stronger winds have a ventilation effect (Fig. 3d). The latter anticorrelation is also detected during the wet season at the suburban site for all studied ions. Meanwhile, RH seems to increase ion concentrations. There is a clear anticorrelation between ammonium and anthropogenic elements at both sites during the wet season (Fig. 3b,c,e,f). Finally, during the wet season with rain events, a positive correlation is found between sulfate and anthropogenic traffic-related elements in PM_10_ (Fig. 3c). This may help support the fact that during the rainy days the traffic and driving conditions get worse causing an increase in the exhaust and non-exhaust traffic emissions. Fuel in Ecuador has a high sulfur content (300–650 ppm), causing high concentrations of traffic emissions. During the wet season, especially during the days with rain events, nitrate and ammonium anticorrelate with anthropogenic elements (Fig. 3c,f). There is an exception for nitrate that positively correlates with S and Fe at the urban site, pointing to the increased traffic emissions during the rainy days.

### Precipitation effect on PM_10_ composition

**Figure 4:**
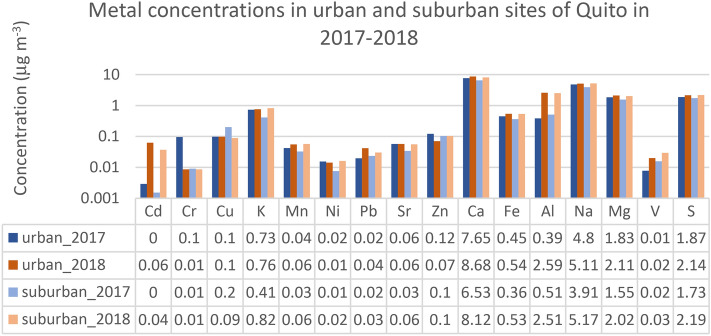
Average concentrations of PM_10_ elements at urban and suburban sites during 2017 (rainier year) and 2018. This figure was produced MS Excel v.10 software^52^.

The results at both sites point to the importance of precipitation for PM_10_. The cumulative precipitation was 1511.8 mm in 2017 and 1,155.4 mm in 2018 at the suburban site. This demonstrates that the total accumulation of precipitation was higher in 2017, with higher maximum hourly precipitation of 44.8 mm (average 0.17 ± 1.18 mm), compared to 37.6 mm (average hourly 0.14 ± 1.07 mm) in 2018. Even bigger difference between yearly cumulative precipitation was found at the urban site, with 1788.8 mm in 2017 and 1,001.1 mm in 2018. Maximum hourly precipitation was close to twice as high in 2017 (40.3 mm) compared to 2018 (22.8 mm). The same pattern was registered for the average hourly precipitation of 0.21 ± 1.38 mm in 2017 and 0.12 ± 0.83 mm in 2018 at the urban site. Significantly stronger rain events at both sites in 2017 might suggest a better PM removal in 2017 from the urban environment^17,20^. This may also help explain lower concentrations in 2017 of many elements coming from natural sources (e.g., K, Ca, Fe, Al, Na, Mg and Mn) and a few anthropogenic elements (e.g., Cd, Ni, Pb, V and S) (see Fig. 4). Interestingly, some metals like Cr and Zn, that are markers for fossil fuels and brake wear^8–10,13^, indicate an increase during the rainier year (Fig. 4). This most likely is due to a worsened combustion efficiency and an increased traffic activity, thus more braking and accelerating. Finally, all studied ion concentrations show an increase during the rainier 2017 year (not shown), pointing to the increased ion formation due to an increase in precursor emissions and gas-to-particle transformation during the events of elevated humidity.

**Figure 5:**
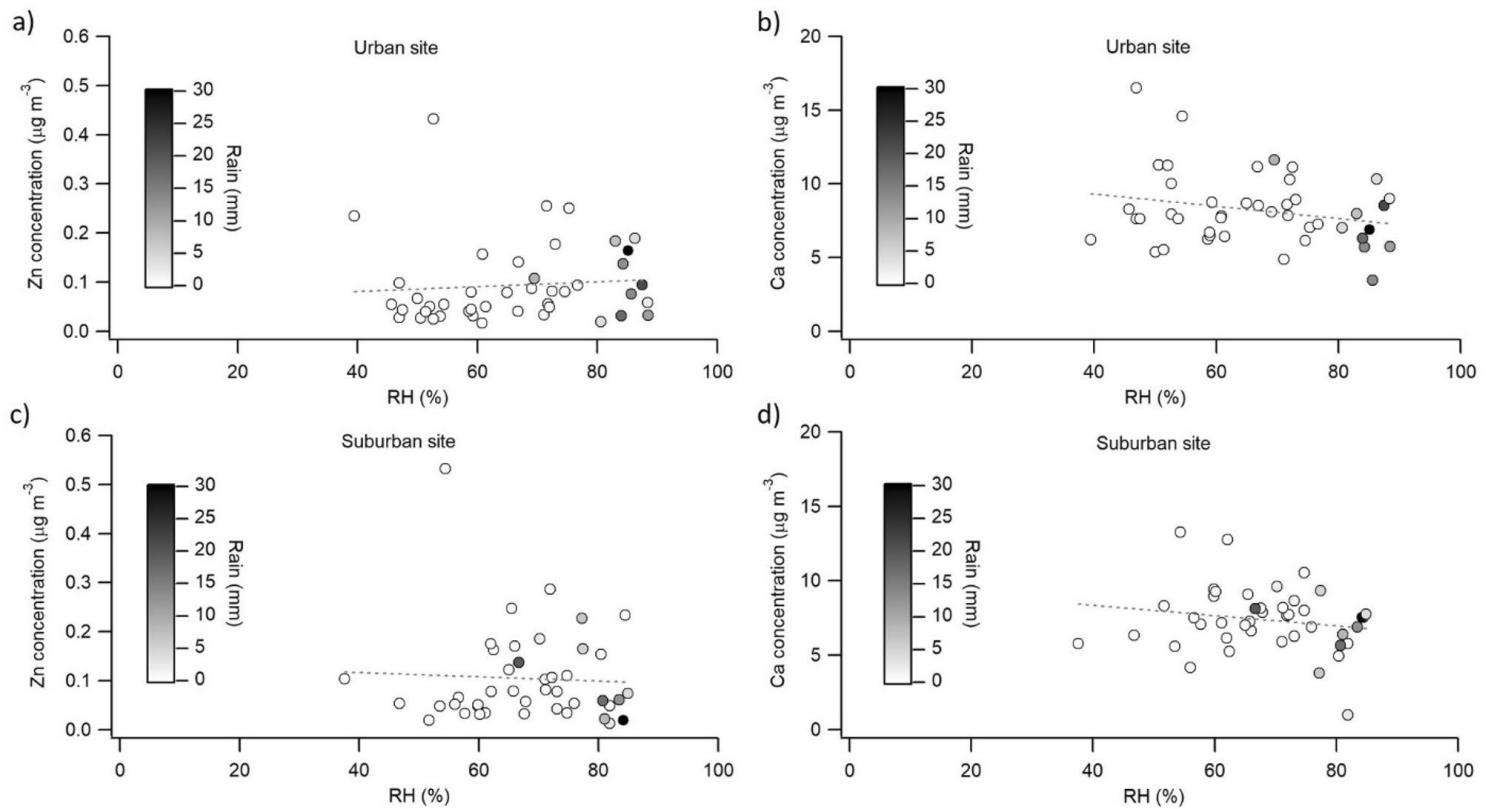
Relative humidity and precipitation effect on concentrations of selected metals in urban (**a**,**b**) and suburban (**c**,**d**) sites during 2017–2018: Zn (**a**,**c**) and Ca (**b**,**d**). This figure was produced using Igor Pro v.8.0 software (WaveMetrics, Inc)^53^.

Contrasted relative humidity and precipitation effects on PM_2.5_ concentrations were found in a previous study performed at two different sites: (1) urban area with busy traffic; and (2) a more industrialised suburban area^37^. It was suggested that the different effect might be due to the difference in sources. For instance, in the less motorized site the RH had a clear negative effect on PM_2.5_ concentrations, while in the highly motorized urban area RH had a positive effect unless there was a strong precipitation event. Thus, the correlation between relative humidity and concentrations of natural (e.g., Ca) and anthropogenic (e.g., Zn) elements of PM_10_ in relation to the strength of precipitation, was investigated (Fig. 5 and Figure B1, Online Appendix B). It can be seen that, in most cases, the concentrations of PM_10_ elements decrease with an increasing RH and also with more accumulation of precipitation (Fig. 5b–d and Figure B1, Online Appendix B). This negative correlation between the concentrations of PM_10_ elements and rainfall intensity was reported by other studies^22^_._ One exception is anthropogenic elements at the urban site, which at increasing humidity and precipitation show an increase in concentrations (Fig. 5a). Several studies show that both RH and elevation enhance particulate emissions from internal combustion engines^15,25,26,73,74^, due to the reduced air-to-fuel ratio of the combust mixture. This, and previously discussed worsened traffic conditions causing more acceleration and braking activities, may explain the increase in the levels of anthropogenic elements in PM_10_ during the wet season in this high elevation city.

### Morphological characterization of PM_10_

**Figure 6:**
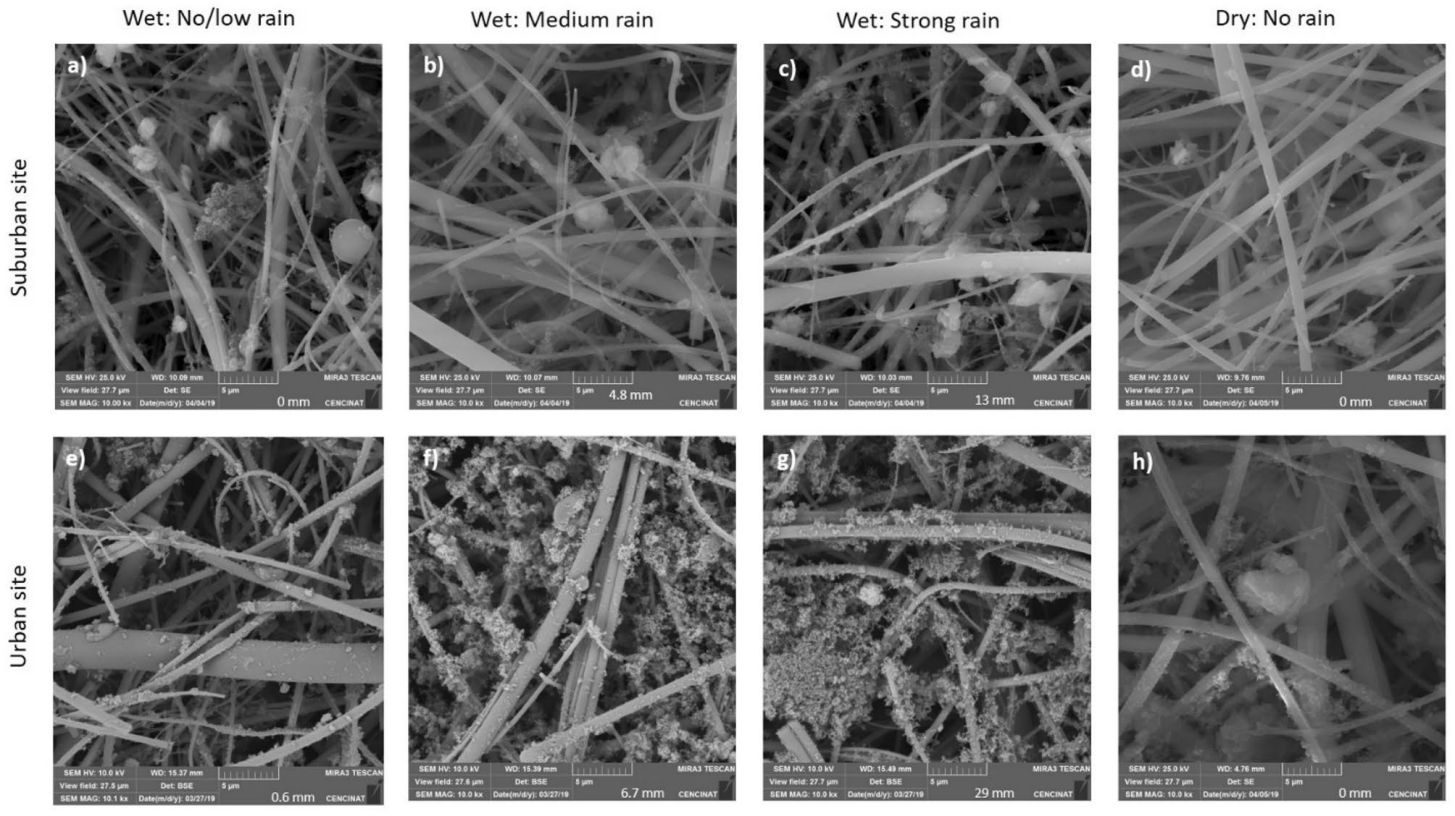
Selected 5 µm resolution scanning electron microscopy (SEM) images of PM_10_ filter samples for the suburban (**a**–**d**) and urban (**e**–**h**) sites in Quito for different meteorological conditions: (**a**) and (**e**) no/low rain during the wet season (0–1 mm); (**b**) and (**f**) medium rain during the wet season (> 1–9 mm); (**c**) and (**g**) strong rain during the wet season (> 9 mm); and (**d**) and (**h**) no rain during the dry season.

We are aware of the limitations of our study, related to the partial chemical categorization of PM_10_ filter samples and reduced sampling resolution. However, we are confident that we have a sufficient set of data for every specific season to help us contribute to the scientific knowledge on the seasonality effect on the composition of PM_10_. The gravimetric results of PM_10_ concentrations were compared with the elemental and ion analysis, and 64% and 72% of the chemical composition was explained for the suburban and urban sites, respectively. We confirm that the biggest mass fraction is occupied by the natural mineral soil-like particles and water-soluble ions, while the rest of it might be other elements and organic and black carbon particles. The latter can be explained by the SEM morphological analysis performed for different days with rain and without rain events during the dry and wet seasons for the suburban and urban sites. Figure 6 shows SEM images of quartz fibre filters after 24-h particle collection. It is observed that many particles are agglomerated and embedded in the fibre filter. Foremost, it can be seen from Fig. 6 that the morphology of PM_10_ is quite different in the two sites. The samples from the suburban site show more isolated and bigger size particles trapped in the filter (Fig. 6a–d) than the samples from the urban site (Fig. 6e–h), which suggests the presence of natural origin particles. However, the presence of individual particles with spherical shape and smooth surface (Fig. 6a) were also observed. These particles are associated with anthropogenic processes^75^ and are indicative of combustion or other high-temperature processes characteristic of industrial activities, such as combustion of mineral coal^76^, or of exhaust emissions from automobiles using gasoline or diesel combustibles^49,77,78^. This type of particles, with the diameters of 3–6 μm, at this suburban site might come from a coal-fired power plant located at around 4 km from the sampling area (Fig. 1). Particles with irregular shapes (length of 6.5–11.5 μm) were also observed (Fig. 6b,c). They can arise from geological material such as road dust, soil, minerals, etc.^78,79^. Meanwhile, the urban site filters are dominated by smaller soot-like particles^80,81^ (Fig. 6e–h) and show agglomerated soot particles, formed through aggregation processes of spherical carbonaceous particles, which support an anthropogenic origin. Moreover, the presence of a greater number of soot agglomerates is evident in the wet season (Fig. 6e–g) compared to the dry season (Fig. 6h), which confirms that the highest concentration of PM_10_ occurs in the wet season (see Table 2). This also helps explain an increase in some anthropogenic origin metals that often stick to the soot particles in the urban environment, usually due to an increase in traffic activity during the rainy days of the wet season. However, as precipitation events get stronger, there is no evident immediate cleansing effect on PM_10_ at the urban site (Fig. 6f,g). On the contrary, the urban site filters seem to get more packed with soot-like particles with increasing accumulation of precipitation. This might be due to the significantly higher relative humidity during the days of rain events (Table 4), which reduces engine combustion efficiency and causes more emissions.

## Conclusions

This, first of its kind, study investigates the chemical elements and water-soluble ions composition of particulate matter with aerodynamic diameters ≤ 10 µm (PM_10_) in the high elevation Ecuadorian capital, Quito (elev. 2,835 m.a.s.l.). Two years (2017–2018) of PM_10_ filter samples show a build-up trend of PM_10_ concentrations during the dry season at the suburban site, due to the increased solar radiation, temperature and wind speed, and the reduced wet scavenging, favouring particle resuspension. Meanwhile, at the densely urbanized site, PM_10_ peaks during the wet season in the days of no rain events. The results at the urban and suburban sites point to the importance of precipitation scavenging of PM_10_.

During the study period, there were some differences registered between the PM_10_ elements of natural and anthropogenic origins. While 2017 was a rainier year, especially at the urban site, some traffic-related metals (Cr and Zn) displayed increased concentrations due to the worsened traffic (more stop-and-go and braking) and combustion conditions. Meanwhile, the elements of a natural origin (e.g., K, Ca, Fe, Al, Na, Mg, Mn) registered decreased levels, indicating the importance of wet scavenging. In addition, in the urban site, most of the elements display higher concentrations during the wet seasons.

Correlation and cluster analyses show the importance of wind speed on the transport of industrial and resuspension of natural pollution at the urban site. Meanwhile, the relative humidity and precipitation help clear PM_10_ but increases PM_2.5_ concentrations at this site. This again confirms the worsened combustion and traffic conditions during the rainy season. The correlation analysis between relative humidity and concentrations of PM_10_ elements in relation to the strength of precipitation shows that, while in most cases humidity and rain reduce the concentrations of PM_10_ elements, the levels of anthropogenic elements at the urban site keep increasing. This could be explained by the fact that RH and elevation enhance particulate emissions from internal combustion engines.

Finally, SEM morphological analysis showed that the urban site filters are dominated by the smaller soot-like particles, especially during the days of increasingly stronger precipitation events. The results of our study are suggesting an alternate effect of precipitation on the levels of particulate urban pollution. Meanwhile, the suburban site filters show more trapping of natural particles and confirm a clearing effect of wet deposition.

## Supplementary information

Supplementary file 1
