# Supplementary material for: Seasonal variations in PM10 inorganic composition in the Andean city
Source: Sci Rep. 2020 Oct 12;10:17049. doi: 10.1038/s41598-020-72541-2 (PMC7550351; doi:10.1038/s41598-020-72541-2)
Supplement: Supplementary file 1 [file 41598_2020_72541_MOESM1_ESM.docx]

**Seasonal variations in PM_10_ inorganic composition in the Andean city**

Rasa Zalakeviciute ^1,2*^, Katiuska Alexandrino^1^, Yves Rybarczyk ^2,3^, Alexis Debut ^4^, Karla Sofia Vizuete Armendariz ^4^, Maria Valeria Diaz Suarez ^5^

^1^ Grupo de Biodiversidad Medio Ambiente y Salud (BIOMAS), Universidad de Las Américas, calle José Queri y Av. de los Granados / Bloque 7, Quito – EC 170125, Ecuador

^2^ Intelligent and Interactive Systems Lab (SI2 Lab) Universidad de Las Américas (UDLA), Quito, Ecuador

^3^ Faculty of Data and Information Sciences, Dalarna University, 791 88 Falun, Sweden

^4^ Centro de Investigación de Nanociencia y Nanotecnología CENCINAT, Universidad de las Fuerzas Armadas ESPE

^5^ Air Quality Monitoring Network, Secretariat of the Environment, Municipality of the Quito Metropolitan District, Calle Rio Coca, Quito – EC 170125, Ecuador

^*^ - corresponding author: [rasa.zalakeviciute@udla.edu.ec](mailto:rasa.zalakeviciute@udla.edu.ec); [rasa.zalake@gmail.com](mailto:rasa.zalake@gmail.com)

**Appendix A**


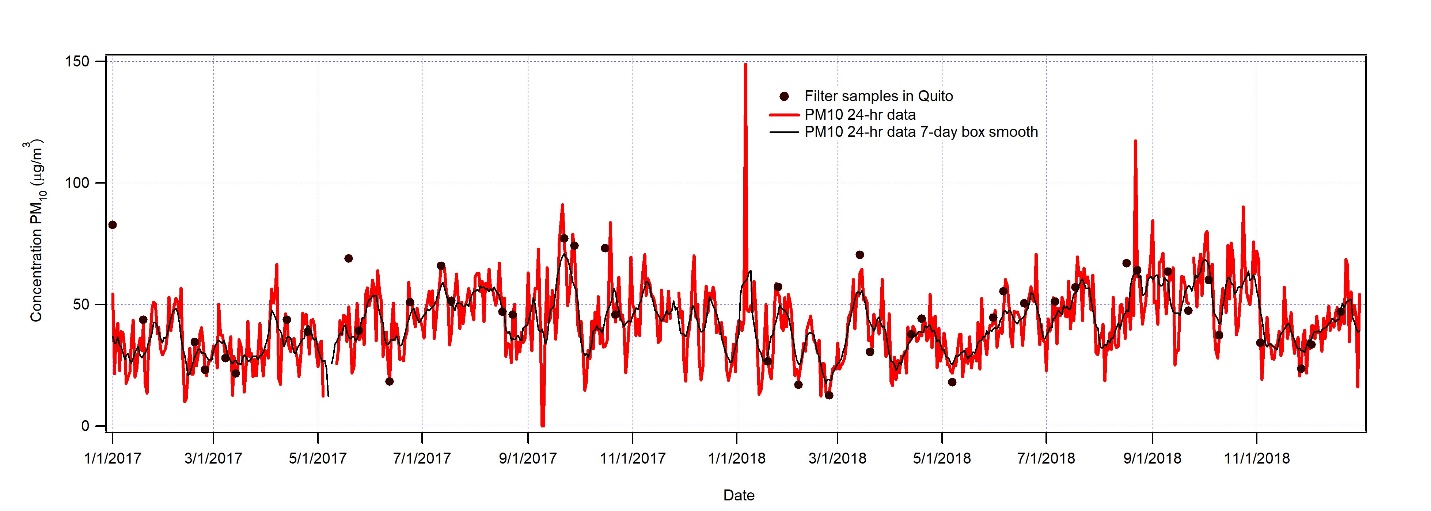


**Figure A1.** 24-hour high volume PM_10_ filter samples (black dots) plotted against a continuous (automatic Thermo Scientific/FH62C14 (PM10 EPA Np. EQPM-1102-150) PM_10_ 24-hour average data (red line) and 7-day box smoothing of the continuous data (black line). Some dissimilarities in the data are expected as the two sets of data are coming from different sites, with available PM_10_ data (e.g., PM_10_ filter samples are collected from the central and southern sites (this work), while the continuous PM_10_ data is collected from two sites 6-15 km north). This figure was produced using Igor Pro v.8.0 software (WaveMetrics, Inc) ^53^.

**Appendix B**


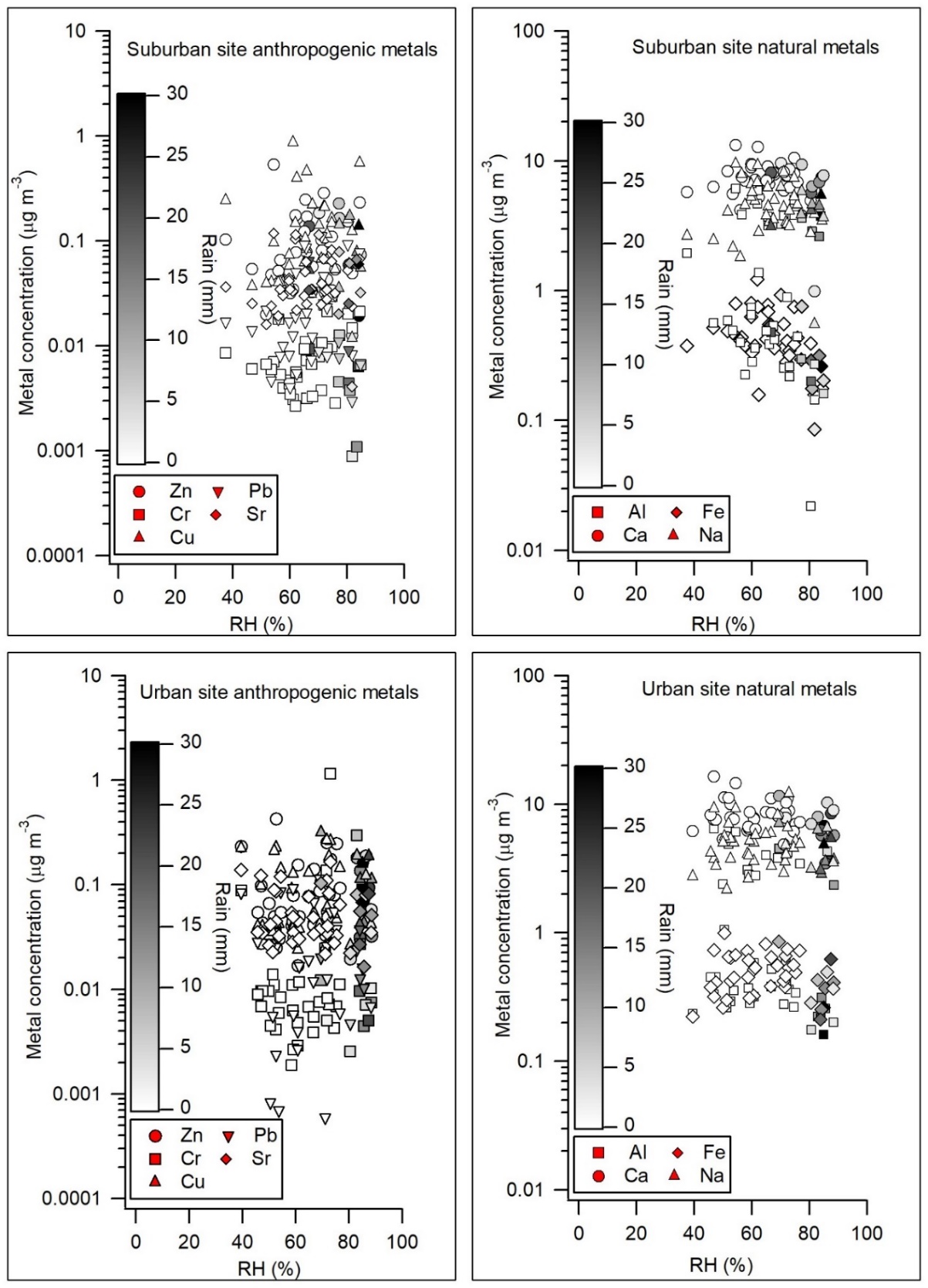


**Figure B1.** Relative humidity and precipitation effect on selected anthropogenic (Zn, Cr, Cu, Pb, Sr) and natural (Al, Ca, Fe, Na) metals in urban and suburban sites during 2017-2018. This figure was produced using Igor Pro v.8.0 software (WaveMetrics, Inc) ^53^.
